# Effectiveness of Social Media Interventions for People With Schizophrenia: A Systematic Review and Meta-Analysis

**DOI:** 10.2196/jmir.5385

**Published:** 2016-04-22

**Authors:** Maritta Välimäki, Christina Athanasopoulou, Mari Lahti, Clive E Adams

**Affiliations:** ^1^ Department of Nursing Science University of Turku Turku Finland; ^2^ Turku University Ηospital Turku Finland; ^3^ Faculty of Health and Well-being Turku University of Applied Sciences Turku Finland; ^4^ Institute of Mental Health Division of Psychiatry University of Nottingham Nottingham United Kingdom

**Keywords:** social media, effectiveness, technology, Internet, Web 2.0, schizophrenia, mental health

## Abstract

**Background:**

Recent studies have shown that people with serious mental disorders spend time online for the purposes of disclosure, information gathering, or gaming. However, coherent information on the effects of social media on treatment for people with schizophrenia is still lacking.

**Objective:**

Our aim was to determine the effects of social media interventions for supporting mental health and well-being among people with schizophrenia.

**Methods:**

A systematic review and meta-analysis were undertaken to determine the effects of social media interventions for supporting mental health and well-being among people with schizophrenia. Ten databases were searched, while search parameters included English-only manuscripts published prior to June 25, 2015. Study appraisals were made independently by 2 reviewers, and qualitative and quantitative syntheses of data were conducted.

**Results:**

Out of 1043 identified records, only two randomized studies of moderate quality (three records, total N=331, duration 12 months) met the inclusion criteria. Participants were people with schizophrenia spectrum or an affective disorder. Social media was used as part of Web-based psychoeducation, or as online peer support (listserv and bulletin board). Outcome measures included perceived stress, social support, and disease-related distress. At 3 months, participants with schizophrenia in the intervention group reported lower perceived stress levels ( *P*=.04) and showed a trend for a higher perceived level of social support ( *P*=.06). However, those who reported more positive experiences with the peer support group also reported higher levels of psychological distress ( *P*=.01).

**Conclusions:**

Despite using comprehensive searches from 10 databases, we found only two studies, whereas numerous reports have been published citing the benefits of social media in mental health. Findings suggest the effects of social media interventions are largely unknown. More research is needed to understand the effects of social media, for users with and without mental illness, in order to determine the impact on mental well-being ofsocial media use as well as its risks.

## Introduction

People with severe mental illness, including schizophrenia, have symptoms that may seriously affect them for life. Evidence-based recommendations of current schizophrenia treatment guidelines include approaches such as medication use, psychotherapy, and family psychoeducational interventions [ 1]. For those with serious mental illness, online interventions have been shown to have the potential to disseminate care, support the patient’s participation in group interactions [ 2], and serve as an alternative for individuals [ 3]. Combining online psychoeducation with various other online tools, including social media, is becoming more popular, and online psychotherapy and videogames have been evaluated for validity and efficacy [ 4].

Recently, the online world has been experiencing a huge growth of user-generated content, which has increased the acceptance of social media [ 5]. Although doubts in professional discourse about Internet use [ 6] or the use of social media in treatment and prevention exist—mostly concerning challenges regarding controlling user behavior, accurately assessing risks, and privacy and confidentiality issues [ 7]—the use of online communities is increasing constantly. More than 64% of Internet users access social media services online, while social networking is one of the most popular ways to contact friends and family [ 8]. Currently, social media users total approximately 2.22 billion [ 9], and 31% of all Internet users spend their time specifically on social networking sites [ 10]. Facebook is the most popular social networking site, with close to 1.6 billion active users as of the fourth quarter of 2015 [ 11]. It has been estimated that in 2017 there will be around 2.39 billion online social network users [ 9]. Given the high penetration rates of social media, it is important to acquire a deeper understanding of the applications of social media in health care [ 12].

Investigating the trend of using social networking interventions is important, as peer-support services are viewed as best practice‒based solid theory and are supported by policy makers [ 13- 15]. Lal and Adair’s rapid review [ 16] indicates that many find an enormous potential for e-mental health to help address the gap between the identified need for services and the limited capacity of resources to provide conventional treatment. Numerous social networking sites have already been developed to change behavior and improve health outcomes [ 17]. Systematic reviews of online interventions have also been conducted, focusing on, for example, online peer support [ 18], Internet support groups for depression [ 19], online communication, social media and adolescent well-being [ 20], social media to address Asian immigrants’ mental health needs [ 21], online and social networking for the treatment of depression in youth [ 22], and social media and suicide prevention [ 7]. Although the results seem promising, the reviews share a concern about poor designs of the original studies with underpowered samples [ 18, 19, 22], mixed findings [ 23], or a lack of intervention studies [ 7]. A Cochrane review by Välimäki et al [ 24] also stresses the importance of high-quality studies in assessing the effects of novel interventions, in particular, the case of virtual reality for people with serious mental illness.

There are few studies that have produced coherent knowledge of the effects of social media use in treatment [ 7]. Research on the effects of online support for people with mental illnesses is even more scarce [ 25], especially when the illness is severe. Although online social media and mobile technology have shown some promise in improving positive psychotic symptoms, hospital admissions, socialization, social connectedness, depression, and medication adherence of people with psychosis, the issues of heterogeneity, poor study quality, and the early state of current research preclude any definite conclusions [ 26]. As far as we are aware, there have been no systematic reviews and meta-analyses to date of social media interventions for people with schizophrenia or similar disorders. Since people with psychosis spend more time in chat rooms or playing online games than other people [ 27], and online social networking can be used for establishing or maintaining relationships or reconnecting with people and online peer support [ 27], this review is important, as it presents a quantitative analysis describing relevant interventions and seeks to determine the effects of social media for supporting mental health and well-being in this group.

## Methods

This systematic review was conducted in accordance with the Preferred Reporting Items for Systematic Reviews and Meta-analysis (PRISMA) [ 28]. The data extraction was in line with the CONSORT-EHEALTH checklist [ 29]. Social media interventions were extracted by using the Template for Intervention Description and Replication (TIDieR) checklist and guide [ 30].

### Search Methodology

Ten databases (PubMed, MEDLINE, Cochrane Database of Systematic Reviews, PsycInfo, CINAHL, JBI, Scopus, ISI Web of Science, SOCIndex, Sociological abstracts) were searched for potentially relevant abstracts. These databases cover a wide range of published research from the field of health and social care. Search parameters included English-only manuscripts published (or in press) prior to June 25, 2015.

The search terms (or equivalent index terms and free-text words) for each of the databases were used to ensure a broad coverage of studies in our review. For example, search terms for schizophrenia included “schizophreni” OR “schizoaffective disorder” OR “schizophrenia” OR “schizophrenia-like illness” OR “schizo-affective disorder” OR “severe mental illness” OR “severe mental illnesses” OR “serious mental illness” OR “schizophrenia and disorders with psychotic features”. Search terms for social media included “social media” OR “Internet” OR “world wide web applications” OR “blogging” OR “blog” OR “wiki” OR “facebook” OR “twitter” OR “youtube” OR “Instagram” OR “web 2.0” OR “chat” OR “chats” OR “chatting.” The detailed terms for each database were searched by the information specialist at the University of Turku. Search terms for each database are presented in Table 1.

**Table 1:** Databases, search terms, and references found on June 25, 2015 (N=1043).

Database	Search terms	N
PubMed	(schizophreni* OR schizoaffective disorder* OR “severe mental illness” OR “severe mental illnesses” OR “serious mental illness” OR “serious mental illnesses” OR “schizophrenia-like illnesses” OR “schizophrenia-like illness” OR “Schizo-affective disorder” OR “Schizo-affective disorders” OR “Schizophrenia and Disorders with Psychotic Features”[Mesh]) AND (“Blogging”[Mesh] OR “Social Media”[Mesh] OR “Internet”[Mesh] OR Social media* OR Wiki* OR Facebook* OR Twitter * OR Youtube* OR Instagram* OR web 2.0 OR blogging OR blog* OR chat OR chats OR chatting*)	324
Ovid Medline	((schizophreni* or schizoaffective disorder* or severe mental illness* or serious mental illness* or schizophrenia-like illness* or Schizo-affective disorder*).mp. or exp Schizophrenia/) and (exp Blogging/ or exp social media/ or exp Internet/ or (Social media* or Wiki* or Facebook* or Twitter* or Youtube* or Instagram* or web 2* or blogging or blog* or chat*).mp.)	166
JBI	(schizophreni* or schizoaffective disorder* or severe mental illness* or serious mental illness* or schizophrenia-like illness* or Schizo-affective disorder*).mp. and (Social media* or Wiki* or Facebook* or Twitter* or Youtube* or Instagram* or web 2* or blogging or blog* or chat*).mp.	8
CINAHL	((MH “Schizophrenia+”) OR schizophreni* OR “schizoaffective disorder*” OR “severe mental illness*” OR “serious mental illness*” OR “schizophrenia-like illness*” OR “Schizo-affective disorder*”) AND ((MH “Social Media”) OR (MH “World Wide Web Applications+”) OR (MH “Instant Messaging”) OR (MH “Blogs”) OR “Social media*” OR Wiki* OR Facebook* OR Twitter* OR Youtube* OR Instagram* OR “web 2.0” OR blogging OR blog* OR chat*)	8
Cochrane	(schizophreni* or schizoaffective NEXT disorder* or severe NEXT mental NEXT illness* or serious NEXT mental NEXT illness* or schizophrenia NEXT like NEXT illness* or Schizo NEXT affective NEXT disorder*) and (Social NEXT media* or Wiki* or Facebook* or Twitter* or Youtube* or Instagram* or web NEXT 2* or blogging or blog* or chat*)	24
PsycInfo	(SU.EXACT.EXPLODE(“Schizophrenia”) OR schizophreni* OR “schizoaffective disorder*” OR “severe mental illness*” OR “serious mental illness*” OR “schizophrenia-like illness*” OR “Schizo-affective disorder*”) AND (SU.EXACT.EXPLODE(“Social Media”) OR “Social media*” OR Wiki* OR Facebook* OR Twitter* OR Youtube* OR Instagram* OR “web 2.0” OR blogging OR blog* OR chat*)	276
Web of Science	(schizophreni* OR “schizoaffective disorder*” OR “severe mental illness*” OR “serious mental illness*” OR “schizophrenia-like illness*” OR “Schizo-affective disorder*”) AND (“Social media*” OR Wiki* OR Facebook* OR Twitter* OR Youtube* OR Instagram* OR “web 2*” OR blog* OR chat*)	121
Scopus	(schizophreni* OR “schizoaffective disorder*” OR “severe mental illness*” OR “serious mental illness*” OR “schizophrenia like illness*” OR “Schizo affective disorder*”) AND (“Social media*” OR Wiki* OR Facebook* OR Twitter* OR Youtube* OR Instagram* OR “web 2.0” OR blog* OR chat*)	84
SOCIndex	(DE “SCHIZOPHRENIA”OR schizophreni* OR “schizoaffective disorder*” OR “severe mental illness*” OR “serious mental illness*” OR “schizophrenia-like illness*” OR “Schizo-affective disorder*”) AND (DE “SOCIAL media” OR DE “BACKCHANNELS (Social media)” OR DE “BLOGS” OR DE “COMPUTER bulletin boards” OR DE “ONLINE chat” OR DE “SOCIAL bookmarks” OR DE “WEB 2.0” OR OR DE “ONLINE comments” OR DE “ELECTRONIC discussion groups” OR “Social media*” OR Wiki* OR Facebook* OR Twitter* OR Youtube* OR Instagram* OR “web 2.0” OR blogging OR blog* OR chat*)	12
Sociological abstract	(SU.EXACT.EXPLODE(“Schizophrenia”) OR SU.EXACT.EXPLODE(“Paranoia” OR “Psychosis” OR “Schizophrenia”) OR schizophreni* OR “schizoaffective disorder*” OR “severe mental illness*” OR “serious mental illness*” OR “schizophrenia-like illness*” OR “Schizo-affective disorder*”) AND (SU.EXACT(“Computer Mediated Communication”) OR SU.EXACT.EXPLODE(“Internet”) OR “Social media*” OR Wiki* OR Facebook* OR Twitter* OR Youtube* OR Instagram* OR “web 2.0” OR blogging OR blog* OR chat*)	20

Some differences between databases and search words used are due to available thesaurus terms in the specific databases, that is, the descriptor/thesaurus term “electronic discussion groups” is used only in SOCIndex, in an attempt to translate the Medical Subject Headings (MeSH) terms used.

### Inclusion and Exclusion Criteria

The review was limited to texts published in English with an abstract available (published on or before June 25, 2015). Participants were people with schizophrenia spectrum disorders. If the study included other participants, data from those people were included only if reported separately. The review topic was limited to studies concerning interactivity and social media. Interactivity refers to user-to-user contact [ 31], such as patients with peers, staff members, or their nearest or public social media. Social media was understood as a broader term including collaborative projects (eg, user-generated content, content communities, content sharing, and social online networking sites) [ 5, 32- 34], social networking (eg, the broader concepts of Health 2.0 and Medicine 2.0) [ 35], or interventions involving Facebook, Twitter, YouTube, Instagram, blogs, Wiki, chats, the Internet, or Web 2.0. The concept was used in the health care domain and targeted adult persons with various schizophrenia spectrum diagnoses. If the study included adolescents, it was included only if the mean age of the participants was over 30 years. Only peer-reviewed, published papers with randomized clinical trials were included.

Studies were excluded if the information and communication technology was used only in interventions for data collection purposes (eg, online surveys, electronic medical records), for patient education without any online social networking or to print the paper material for participants. Further, papers describing the design or the development process of the social media intervention, theoretical or methodological papers, books or book chapters, letters, dissertations, editorials, or study protocols were excluded.

In the case of multiple publications from the same study, we combined data to avoid double counting. When required, we contacted the corresponding author to acquire more detailed data if the data we were interested in were not available in the publication.

### Study Identification

**Figure 1:**
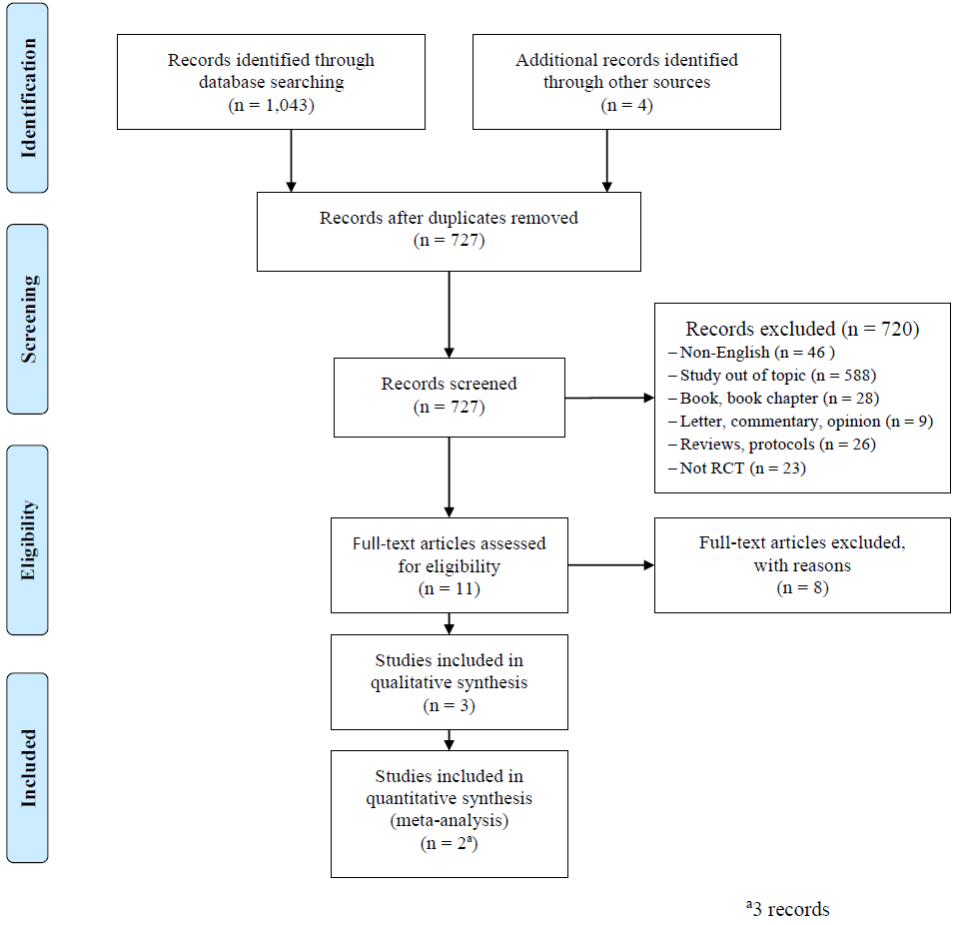
PRISMA flow diagram outlining the review process.

Out of 1043 hits, duplicates across all databases were removed, leaving us with 727 abstracts. First, 2 authors (MV, ML) screened titles and abstracts independently for eligibility; ineligible hits were excluded (n=720). Second, we found 4 additional papers when an additional hand search of lists of references was conducted. Third, 11 full papers were obtained and screened by the 2 authors for the inclusion and exclusion criteria. Finally, eight papers had to be excluded (see Table 2). The systematic review and meta-analysis were conducted on the three retrieved papers (two studies). In cases of discrepancy concerning the decisions made between the reviewers, the papers were discussed until a consensus was reached with the support of CA. Figure 1outlines the search process of the literature, according to PRISMA [ 28].

Excluded studies (n=8) are categorized based on Higgins [ 36]. The specific reasons for exclusion are described in Table 2[ 37- 44].

**Table 2:** Description of excluded studies.

Study	Description	Reason for being excluded ^a^
Kilbourne et al 2013 [ 37]	Cluster randomized adaptive implementation trial comparing a standard versus enhanced implementation intervention to improve uptake of an effective re-engagement program for people with serious mental illness	Allocation: cluster randomization
		Participants: people with serious mental illness
		Intervention: no social media
Kim & Stout 2010 [ 38]	The effects of interactivity on information processing and attitude change: implications for mental health stigma	Allocation: non-randomized
		Participants: undergraduate students
		Intervention: no social media
McFarlane et al 1995 [ 39]	Multiple-Family Groups and Psychoeducation in the Treatment of Schizophrenia	Allocation: randomized
		Participants: people with schizophrenia, schizoaffective disorder, or schizophrenia form disorder (DSM-IH-R)
		Intervention: no social media
Sicilia et al 2005 [ 40]	Effects of interactivity in a website—The moderating effect of need for cognition	Allocation: non-randomized
		Participants: consumers
		Intervention: no social media
Spinzy et al 2012 [ 41]	Does the Internet offer social opportunities for individuals with schizophrenia? A cross sectional pilot study	Allocation: non-randomized
		Participants: people with psychotic disorders and affective disorder, anxiety disorders F20-F48 (ICD-10)
		Intervention: no social media
Steinwachs et al 2011 [ 42]	A Web-based program to empower people with schizophrenia to discuss quality of care with mental health providers	Allocation: non-randomized
		Participants: people with schizophrenia (no classification code specified, ICD-10 or DSM-V)
		Intervention: no social media
van der Krieke et al 2012 [ 43]	Usability evaluation of a Web-based support system for people with schizophrenia diagnosis	Allocation: non-randomized
		Participants: people with schizophrenia or a related psychotic disorder (eg, schizo-affective disorder, schizophreniform disorder, schizotypal disorder) (no classification code specified, ICD-10 or DSM-V)
		Intervention: no social media
van der Krieke et al 2013 [ 44]	A Web-based tool to support shared decision making for people with a psychotic disorder: Randomized controlled trial and process evaluation	Allocation: randomized
		Participants: people with non-affective psychosis (DSM-IV)
		Intervention: no social media

^a^ICD-10=International Classification of Diseases, 10 ^th^Revision; DSM-IV=Diagnostic and Statistical Manual of Mental Disorders, 4 ^th^Edition.

### Data Extraction and Analyses

We created a specific data extraction matrix to collect information. Where possible, data extraction was based on the CONSORT-EHEALTH checklist [ 29]. First, characteristics of the studies were described (country of origin, purpose of the study, setting of patient recruitment, patient group, inclusion criteria, number of patients enrolled, follow-up period, and number of drop-outs). Second, social media interventions were extracted by using the TIDieR checklist [ 30] and guide. The matrix used in our extraction was based on a 12-item checklist and was constructed with the following information: brief name, why (rationale or theory), what (materials, procedures), who provided intervention, how, where, when and how much, tailoring, modifications, and how well (planned, actual) [ 30]. Third, outcomes and instruments used in each study were identified. Fourth, reasons for exclusion of studies were described.

For narrative analysis, data on each included study were entered into the data extraction matrix; each study was treated as a separate case. Descriptive characteristics of the studies were categorized manually. In addition, social media usage and stress after social media use were described. Quantitative analysis was undertaken using the Review Manager 5.3 [ 45], which is the software used for preparing and maintaining Cochrane Reviews. The data analysis was divided into two phases. First, the analysis explored the descriptive characteristics of the individual studies included. Second, for continuous outcomes we estimated the mean difference between groups. When similar scales, such as symptom checklists, were used, we presumed there was a small difference in measurement tools and combined the measurements. This decision was made to answer the overall question of whether there is evidence that social media can be an effective intervention among people with schizophrenia [ 36]. In this approach, standard deviations were used together with the sample sizes to compute the weight given to each study. Random effect was used instead of fixed effect because random effect allows the outcomes of studies to vary more than fixed effects. In other words, random effects can be seen to be a more natural way of explaining outcomes [ 46]. Heterogeneity was checked by calculating I ^2^statistic. Where the I ^2^estimated was greater than, or equal to 50%, it was interpreted as indicating the presence of high levels of heterogeneity [ 36].

### Quality Assessment

Quality of the included studies was assessed based on the Cochrane Collaboration’s tool for assessing risk of bias [ 36]. This tool assesses the possible bias in randomized controlled trials including random sequence generation, allocation concealment, blinding of participants and blinding of outcome assessment, incomplete outcome data, selective reporting, and other possible biases. The assessment was undertaken independently by 2 reviewers (ML, MV). Again, in cases of disagreement, differing views were discussed, decisions were documented decisions, and if necessary, authors of studies were contacted for clarification.

The data were extracted from all included studies. Data presented only in graphs and figures were extracted. In case of any discrepancy, the solution was based on discussion with the third author (CA).

## Results

### Characteristics of the Studies

Both studies were conducted in the United States. Participants were recruited from out-patient facilities [ 47, 48] or using websites and e-newslists [ 25]. Rotondi’s study [ 47, 48] included people with schizophrenia or schizoaffective disorder (Diagnostic and Statistical Manual of Mental Disorders IV criteria), with a mean age of 38 years (SD 11). Ten participants were male (32%), and 15 were white (48%). In the study by Kaplan et al [ 25], the participants were diagnosed with schizophrenia spectrum or affective disorder, their mean age was 47 years, one third were male (n=102), and 87% were white (n=260). Information about the studies’ characteristics and methodology are presented in Table 3[ 25, 47, 48].

### Intervention Characteristics

Interventions included interactivity and social media. The participants in the intervention group used peer-support forums [ 25, 48], while the participants in the control group [ 48] underwent treatment as usual or were under waiting list control conditions [ 25]. In Rotondi at al’s study [ 48], “the usual care” was not further described. In Kaplan’s study [ 25], those who were assigned to the waitlist control condition were asked to refrain from using all forms of Internet peer support for the duration of their participation in the study. Interventions are described in detail in Table 4[ 25, 48].

### Outcome Measures

The studies included specific outcomes: symptoms, knowledge level, recovery, quality of life, empowerment, social support, or stress. A variety of instruments were used in each study. The outcomes and instruments used in the studies are listed in Table 5[ 47- 58].

In addition, in Kaplan et al’s study [ 25], participants’ experiences with the online group were assessed with the 7-item measure (Online Group Questionnaire [OGQ], adapted from Chang et al [ 59]). The OGQ contained questions about how often they thought the discussion topic was relevant, whether they felt supported by the group members, or they were satisfied with the group overall.

**Table 3:** Study characteristics.

Categories	Rotondi et al 2005, 2010 [ 47, 48]	Kaplan et al 2011 [ 25]
Country of origin	United States	United States
Purpose of the study	To examine use of websites and home computers to deliver online multifamily psychoeducational therapy to people with schizophrenia (and their informal supports).	To test the effects of unmoderated, unstructured Internet peer support for people with psychiatric disabilities.
Setting of patient recruitment	Community mental health centers and inpatient units.	Websites, e-newslists, study advertisements via mental health provider agencies and programs.
Inclusion criteria	People diagnosis of schizophrenia or schizoaffective disorder, 14 years of age or older, one or more psychiatric hospitalizations or emergency department visits within the previous 2 years, ability to speak and read English, living in the community at the time of study entry, and absence of physical limitations that would preclude using a computer.	People diagnosed with a schizophrenia spectrum or an affective disorder, access to both a computer and the Internet, no use of Internet peer support in the past year, US resident, fluent in English.
Randomization	Randomly assigned	Randomly assigned, block randomization
Number enrolled	31	300
Follow-up period	3, 6, and 12 months	4 and 12 months
Number leaving early	1	41

**Table 4:** Description of the interventions (modified based on TIDieR checklist and guide).

Categories	Rotondi et al 2010 [ 48]	Kaplan et al 2011 [ 25]
Brief name	Schizophrenia Online Access to Resources (SOAR) intervention, specifically made for the study (the telehealth group).	Peer support Listserv or Peer support bulletin board. A Listserv and bulletin board were specifically made for the study.
Rationale/theory	Designed to provide key elements of family psychoeducation: empathetic engagement of participants, education about the illness and treatments, a supportive safety net, and coping strategies. Previous theoretical and empirical work: meeting and individual’s needs can reduce stress, promote better adaption to illness-related difficulties, and improve outcomes; promotion of self-efficacy, self-management, and problem solving.	Participation to the online support group would result in improved well-being and decreased distress.
Materials	Computer access to the Internet via a dial-up modem and local Internet service provider, Schizophrenia Guide website/software.	Computer and Internet access. Participants received a message describing how their participation was to occur, how it worked, security information, warnings, advice, resources, and contact information.
Procedures	Participants received dial-up Internet access and a computer (if not having already). They were granted access to the following information and services via the “Schizophrenia Guide Web Site”: 1. 3 online therapy groups, 2. Ask Our Experts Your Questions, 3. Questions and Answers Library, 4. Educational and Reading Materials; and 5. What’s New. Participants were interviewed and provided their subjective evaluations of the website, several aspects of social support were also accessed.	1. Participants assigned to the experimental Listserv group communicated anonymously with each other using a group distribution email list; 2. Participants in an experimental peer support bulletin board group were given instructions on how to use the bulletin board. The content of Listserv and the bulletin board were entirely peer directed.
Providers	Project team members answered the questions of “Ask Our Experts Your Questions” module; therapy forums were facilitated by experienced mental health professionals (master of social work and PhD clinicians) trained in the monitoring and management of Web-based interventions; trained interviewers collected self-report data from participants.	Interventions were directed for study participants only and not facilitated by staff. Research staff was available for technical help.
How	The website provided 3 therapy forums (one for patients, one for support persons, one for both groups), a capability for asking questions of and receiving answers from a project team within 24-48 hours. The therapist emphasized discussions that focused on problem solving, and interacting with peers to develop a supportive forum where members could work together to address problems.	With both the Listserv and bulletin board, individuals communicated anonymously with one another using a group distribution email list specifically created for the group. The participants were encouraged to read and respond to email messages.
Where	The participants worked at home (Pittsburgh area, Pennsylvania, United States) and had access to the SOAR intervention via a desktop icon.	The participants worked at home and had access to Listserv or the bulletin board.
When and how much	Telehealth participants attended a joint, 4-hour workshop. Participants were in the study for up to 1 year.	Participants were in the study for 12 months.
Tailoring and modifications	N/A	N/A

**Table 5:** Outcomes and instruments used in the studies.

Outcomes	Rotondi et al 2005, 2010 [ 47, 48]	Kaplan et al 2011 [ 25]
Symptoms	Scale for the Assessment of Positive Symptoms [ 49] ^a^	The Hopkins Symptom Checklist [ 50]
Knowledge level	Knowledge About Schizophrenia Instrument [ 51]	-
Recovery	-	The Recovery Assessment Scale [ 52]
Quality of life	-	The Quality of Life Interview, QoL [ 53]
Empowerment	-	The Empowerment Scale [ 54]
Social support	Perceived social support [ 55- 58]	The Medical Outcome Study [ 58]
Stress	Self-rated stress [ 55- 58]	-

^a^Information not available.

### Narrative Analysis

An analysis of social media usage was reported in both studies [ 25, 48]. The time that people with schizophrenia spent online on the SOAR website was, in total, 43,789 minutes (730 hours); the time involved 47,630 page views. The average time spent on the SOAR website was 46 hours, with an average of 2977+/-4.5 page views [ 48]. The users of the SOAR website asked on average 113 questions and read 69 articles. They used an average of 124 minutes asking questions and 1643 minutes for reading articles [ 48].

Analysis of engagement in social media forums used in the studies [ 25, 48] showed an engagement of people with schizophrenia. They were active in therapy forums during the 3300 sessions [ 48]. They also sent 11,105 messages in different bulletin board forums [ 25].

Kaplan et al [ 25] categorized the participants into “high” and “low” dose participants. The participants categorized in the “high participation” group (57/185) reported having read the messages at least weekly and sent at least 5 messages at the 12-month post baseline point. People in this “high participation” group showed significantly higher distress levels at 4 months and at 12 months, while participants in the “low participation” group reported less distress at 12 months than at baseline. Further, the participants were grouped into those who had a “positive online experience” (OGQ scores ≥3) and those with a “less positive experience” (OGQ scores <3). People who reported positive experiences using social media forums (n=90) were significantly more distressed than participants in the less positive experience group [ 25].

### Effectiveness of the Social Media Interventions

**Figure 2:**
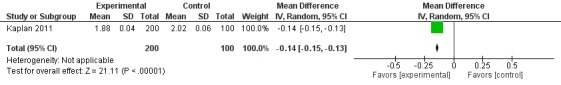
Positive symptoms for experimental and control groups by 6 months or less.

Meta-analysis was performed on both the Kaplan et al [ 25] and Rotondi et al [ 48] studies. In Kaplan et al’s study [ 25], comparisons for symptoms less than 6 months from baseline showed some improvement in the social media intervention group ( *P*<.001, median -0.14, 95% CI -0.15 to -0.13) (see Figure 2). The Rotondi et al study [ 48] did not provide the information required regarding symptoms, thus meta-analysis was not performed.

**Figure 3:**
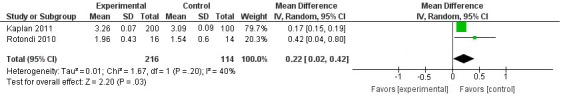
Social support for experimental and control groups by 6 months or less.

**Figure 4:**
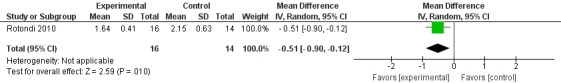
Stress for experimental and control groups by 6 months or less.

Comparisons for social support after 6 months from baseline [ 25, 48] showed some improvement in the treatment as usual group ( *P*=.03, median 0.22, 95% CI 0.02-0.42) (see Figure 3). When self-rated stress was compared 6 months from baseline, Rotondi et al [ 48] reported some effects in the social media intervention group ( *P*=.01, median -0.51, 95% Cl -0.90 to -0.12) (see Figure 4).

**Figure 5:**
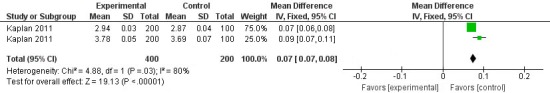
Self-management for experimental and control groups by 4 and 12 months (total score).

**Figure 6:**
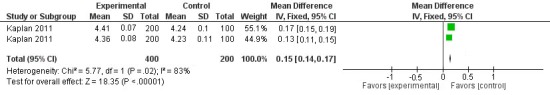
Quality of life for experimental and control groups by 4 and 12 months (total score).

Regarding self-management, Kaplan et al [ 25] compared self-management between groups after 4 and 12 months from baseline. They found that the treatment as usual group was slightly more effective than the social media group ( *P*<.001, median 0.07, 95% CI 0.07-0.089) (see Figure 5). Moreover, Kaplan et al [ 25] compared quality of life after 4 and 12 months from baseline and reported that participants in the social media group had significantly higher QoL scores than participants in the control group ( *P*<.001, median 0.15, 95% CI 0.14-0.17) (see Figure 6).

### Assessment of Methodological Quality

**Figure 7:**
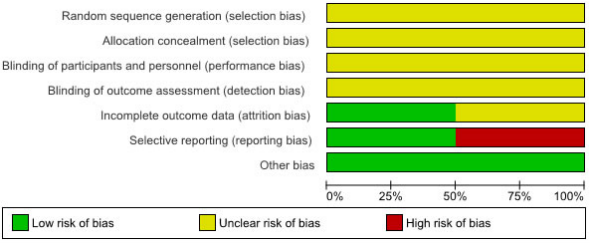
Risk of bias assessment.

The methodological quality of the two studies varied. Incomplete details in reporting the sequence generation and allocation concealment, decreases the methodological quality of both studies. Neither study was blinded, nor was an attempt made at blinding because of the nature of the intervention. Selective reporting may be possible as study protocols were not available. Intention-to-treat analyses were used in both the Kaplan [ 25] and Rotondi [ 47, 48] studies. More details about possible risk of bias can be found in Table 6and Figure 7.

**Table 6:** Outcomes and instruments used in the studies.

Bias	Rotondi et al 2005, 2010 [ 47, 48]	Kaplan et al 2011 [ 25]
Random sequence generation (selection bias)	Randomly assigned. No further details.	Block randomization (nine in each block). No further details.
Allocation concealment (selection bias)	No further details.	No further details.
Blinding of participants and personnel (performance bias)	Non-blinded interviews. No further details.	No further details.
Blinding of outcome assessment (detection bias)	No further details.	Survey conducted using self-report measures. No further details about blinding of outcome assessment.
Incomplete outcome data (attrition bias)	Protocol published in 2003 NCT00051233, no outcomes provided.	No available study protocol.
Selective reporting (reporting bias)	Perceived social support [ 55- 58].	Missing outcome data balanced in numbers across 3 groups. Missing data have been inputted. Retention rate varies slightly (11-18%).

## Discussion

### Principal Findings

To our knowledge, this is the first systematic review and meta-analysis on the effects of social media interventions for people with schizophrenia. Our search strategy allowed us to capture and screen a large number of studies and explore their characteristics, interventions, outcome measures, and quality. Only two studies (three records) fulfilled the criteria and were included, while both showed a strong engagement in social media forums. However, social media use was found to be generally less effective than treatment as usual. Nonetheless, there is not enough evidence to arrive at a definite conclusion.

Existing trials suggest that people with schizophrenia are active in therapy forums and on bulletin boards. They have high participation in social media forums, similar to the general population [ 8]. These findings are in line with previous research, which reports use of forums and online chats by people with psychosis and suggests that such media could play a role in reducing the risk of isolation [ 27]. However, people who were in the “high participation” group showed significantly higher distress levels at 4 and 12 months compared to those in the “low participation” group. Also, those who reported positive experiences using social media forums were more distressed than participants in the negative experience group. As Kaplan et al [ 25] discussed, some clinicians fear that patient participation in online peer support without professional moderation may cause harm by fostering anxiety. Whether these findings really demonstrate this requires more studies.

After comparing the group allocated to social media with treatment as usual, social support, self-management, and quality of life ratings were better for those in treatment as usual. However, another recent systematic review indicated that positive psychotic symptoms, hospital admissions, socialization, social connectedness, and medication adherence have the potential to improve via online and mobile-based interventions for people with psychosis [ 26]. Thus, although based on our review, it appears that treatment as usual might be more effective in many aspects than treatment through social media use, other systematic reviews show that online and mobile-based interventions have a positive impact [ 26]. In our review, social media was part of wider intervention; it was not a pure intervention used for this patient group. Therefore, it would be useful to further investigate the engagement and effects of new technologies, the Internet, and their elements, especially social media, on people with mental disorders, in order to clarify their potential effectiveness for this population.

### Limitations

There are limitations to our review. Only two studies fulfilled the inclusion criteria. Using English language studies might have resulted in our results being biased toward Western countries. It is possible that analysis of studies produced in languages other than English could yield different findings, although we think this unlikely. With both studies originating from the United States, it is unclear if the same findings would be reflected in other countries or cultures. The methodological quality of the included studies was assessed, and we identified a high risk of reporting bias because of missing outcomes or non-availability of study protocols.

### Conclusion

Our findings suggest the effects of social media interventions are largely unknown. Use of social media forums is ubiquitous and increasing, but the relation between social media and mental health is complex [ 60], not well understood, and potentially detrimental. Thus, we suggest that this is reason enough to support further investigation. Emerging evidence suggests that online social networking can be related to major mental health problems such as depression [ 60], but at the same time online and mobile-based interventions for people with psychosis seem to improve depression [ 26]. Given the constant increase of social networking sites, it is understandable that recent studies have identified the need for exploring such sites and mental health [ 12, 34]. Future research should comprehensively assess social media use for people with mental illness to determine the impact of mental well-being for social media use, as well as its risks. In these studies, interventions should be simple and usable to ensure patient engagement. Before suggesting specific social media interventions, however, factors and elements that may foster anxiety and distress by the use of social media should be identified. This knowledge would be an important resource for those who develop and evaluate mental health interventions involving social media, by knowing what elements to avoid in order to make patients’ engagement more pleasant and less distressful. It is also obvious that there is an absence of reliable data coming from high-quality studies to help draw clear conclusions. Thus, robust reporting of the outcome assessment and the study protocol are essential for increasing the quality of studies in this emerging field.

## Abbreviations

CONSORT-EHEALTH: Consolidated Standards of Reporting Trials of Electronic and Mobile Health Applications and onLine TeleHealth
DSM: Diagnostic and Statistical Manual of Mental Disorders
ICD-10: International Statistical Classification of Diseases, 10th Revision
MeSH: Medical Subject Headings
OGQ: Online Group Questionnaire
PRISMA: Preferred Reporting Items for Systematic Reviews and Meta-analysis
QoL: Quality of Life
SOAR: Schizophrenia Online Access to Resources
TIDieR: Template for Intervention Description and Replication
